# Transgenic Mouse Studies to Understand the Regulation, Expression and Function of the Testis-Specific Protein Y-Encoded (TSPY) Gene

**DOI:** 10.3390/genes1020244

**Published:** 2010-08-18

**Authors:** Stephanie Schubert, Jörg Schmidtke

**Affiliations:** Institute of Human Genetics, Hannover Medical School, D-30625 Hannover, Germany; E-Mail: schmidtke.joerg@mh-hannover.de

**Keywords:** testis-specific protein, Y-encoded (TSPY), TSPY-like proteins, male meiosis, Gonadoblastoma oncogene, germ cell tumor stem cells, cell cycle regulation, transcription regulation, neural functions, tumor suppressor, sexual dimorphisms

## Abstract

The *TSPY* gene, which encodes the **t**estis-**s**pecific **p**rotein, **Y**-encoded, was first discovered and characterized in humans, but orthologous genes were subsequently identified on the Y chromosome of many other placental mammals. TSPY is expressed in the testis and to a much lesser extent in the prostate gland, and it is assumed that TSPY serves function in spermatogonial proliferation and/or differentiation. It is further supposed that TSPY is involved in male infertility and exerts oncogenic effects in gonadal and prostate tumor formation. As a member of the TSPY/SET/NAP protein family, TSPY is able to bind cyclin B types, and stimulates the cyclin B1-CDK1 kinase activity, thereby accelerating the G_2_/M phase transition of the cell cycle of target cells. Because the laboratory mouse carries only a nonfunctional Y-chromosomal *Tspy-ps* pseudogene, a knockout mouse model for functional research analyses is not a feasible approach. In the last decade, three classical transgenic mouse models have been developed to contribute to our understanding of TSPY regulation, expression and function. The different transgenic mouse approaches and their relevance for studying *TSPY* regulation, expression and function are discussed in this review.

## 1. Introduction

The human *TSPY* cluster on the human Y chromosome is unique because it represents the largest and most homogenous protein-coding tandem array in the human genome [1]. The major cluster is located close to the centromere on Yp11.2, where each 2.8 kb *TSPY* copy is embedded in a single 20.4 kb DYZ5 tandem repeat array unit [2,3]. While many MSY genes degenerate during evolution due to accumulation of deleterious mutations as a consequence of their suppressed recombination with their X-homologs [4], others avoid degeneration by being present in multiple, nearly identical copies, thereby having the chance to compensate the effects of null alleles via unequal sister chromatid exchange or Y-Y gene conversion [1,5]. This strategy is also applied to the *TSPY* gene, being organized in multiple copies and functionally conserved in primates and cattle [6,7,8,9]. In healthy men, *TSPY* copy number can vary between individuals, ranging from 23 to 64 copies per genome, including functional members and pseudogenes [10]. Functional *TSPY* copies can differ from one another by up to 1% in coding sequences and promoter regions [5]. 

The *Tspy* organization in murid rodents is quite different, in that only single-copy *Tspy-ps* pseudogenes** exist on the Y chromosome in species of the subgenus *Mus* (including the laboratory mouse) [11,12], and in the Mongolian gerbil [13], while several *Apodemus* species and the rat possess only one functional *Tspy* gene. Similar to the human and bovine situation, repetitive functional *Tspy* genes persist on the Y chromosomes of *Mus platythrix* and the Syrian hamster [12,13].

In humans, TSPY is mainly expressed in fetal and adult testis, where its expression is limited to gonocytes and prespermatogonia during fetal and neonatal development, and restricted to spermatogonia, primary spermatocytes and round spermatids in adult testis [14,15,16]. TSPY is also expressed, although much more weakly, in epithelial cells of the prostate gland [17], and some *TSPY* ESTs derived from the medulla of human brains (BX281192, B1828033) have also been reported in databases [16]. Recently, *TSPY* has been classified as a tissue-enriched gene that is highly expressed in a specific organ (testis) and is not expressed, or is expressed at much lower levels, in other tissues [18]. Northern blot and RT-PCR analyses have shown that TSPY is not transcribed in human organs such as heart, lung, liver, spleen, small intestine, kidney, thymus and in leukocytes, however other tissues have not been analyzed up to now [7,19,20].

TSPY expression pattern within the testis as well as its homology to other cyclin B binding TSPY/SET/NAP protein family members point to a role in germ cell proliferation and/or meiotic differentiation. What diverse function(s) TSPY may fulfill in spermatogonial proliferation, differentiation and male meiosis within the testis, and whether it also has a physiological function in the prostate, is currently unknown. It was shown in stably transfected human HeLa and mouse NIH3T3 cells that TSPY could mediate proliferative properties by enhancing the G_2_/M-phase transition of the cell cycle [21]. This effect is directly mediated by the binding of TSPY to the cyclin B1-CDK1 complex, and enhancing its kinase activity [22]. A yeast-two hybrid approach recently identified the eukaryotic translation elongation factor 1 alpha (eEF1A), which is essential for the elongation phase during protein translation, as a second binding partner for TSPY [23]. The findings of the latter study point to a possible function of TSPY in gene transcription and protein translation by interaction with eEF1A. 

TSPY expression is up-regulated in testicular seminoma [24], some non-seminomatous germ cell tumors [25], carcinoma-*in situ* (CIS) [15,26] and gonadoblastoma [27,28,29], and the *TSPY* gene is regarded as the candidate for *GBY*, the elusive gonadoblastoma locus on the human Y chromosome that is thought to have a normal function in the testis but to act as an oncogene in the dysgenetic gonad of XY-sex-reversed and intersex humans [30]. TSPY is also strongly expressed in some somatic tumors, including prostatic carcinoma, hepatocellular carcinoma and melanoma [17,31,32]. However, up to now there is no evidence for a causative role of TSPY in gonadal or somatic tumorigenesis.

Transgenic mice are a powerful tool for promoter, expression and functional research analyses of a specific gene *in vivo*, and since the first use of the classical pronuclear injection technique in 1980, thousands of transgenic mouse lines have been generated [33,34]. The evolutionary knock-out of the *Tspy-ps* in species of the subgenus *Mus*, including the laboratory mouse [11,12,35], has enabled the generation of different transgenic mouse models for studying human *TSPY* regulation, expression and function *in vivo*.

## 2. TSPY transgenic mouse models

### 2.1. Transgenic mouse line TSPY-TAg23: SV40 large and small TAg under the control of a 1.3 kb putative TSPY promoter fragment

#### 2.1.1. Generation of *TSPY-TAg* transgenic mice to investigate the susceptibility of spermatogonia to malignant transformation

Many studies have used transgenic mouse models expressing the viral oncogene simian virus 40 (SV40) large tumor antigen (TAg) under the control of a tissue- or cell-specific promoter for targeted tumor formation, in order to generate suitable animal models for specific types of cancer that can be used to investigate the molecular mechanisms in cellular transformation and evaluate new therapies in oncogenesis [36,37]. It is well known that large tumor antigen TAg encoded by SV40 contributes to cell transformation, in part by binding to and blocking the functions of the tumor suppressors pRb and p53 [38,39]. 

The ability to express SV40 TAg in specific cell types by the use of a cell-specific promoter could be a powerful tool to determine the susceptibility of testicular germ cells to transformation by SV40 TAg at various stages of differentiation. However, this strategy is also limited by some facts. Mice are not commonly susceptible to testicular type-II germ cell tumors (seminomatous, nonseminomatous tumors) and to spermatocytic seminomas, and only testicular teratomas arise spontaneously in mice of the strain 129 with an incidence of 1-8%, depending on the substrain [40,41,42]. Although it is commonly supposed that a delayed or blocked maturation of a primitive germ cell, such as a primordial germ cell (PGC) or gonocyte could be the initial step that leads to formation of most testicular germ cell tumors, the cellular origin of the bulk of testicular germ cell tumors is still unknown [40]. Therefore, generation of transgenic mice in a defined genetic background expressing TAg in early male germ cells such as PGCs or gonocytes could offer useful information about the cellular origin of testicular germ cell tumors, and uncover the molecular mechanisms that lead to tumor formation. 

The first *TSPY* transgenic mouse line (*TSPY-TAg23*) was published in 2003, and was generated in an attempt to investigate the susceptibility of mouse spermatogonia to malignant transformation [43]. This transgenic line was founded on fertilized one cell embryos of FVB mice and was produced using the viral oncoprotein simian virus 40 (SV40) large and small T antigen (TAg), driven by a 1.3 kb putative human *TSPY* promoter fragment. Five founder transgenic mice were generated via pronucleus injection from which only one male (*TSPY-TAg23*) could be out-crossed with FVB wild-type mice and transmitted the transgene to its progeny, thereby founded the transgenic line *TSPY-TAg23*.

#### 2.1.2. kb of a *TSPY* promoter is insufficient to direct a proper tissue-specific expression pattern of TAg in transgenic mice

In 2003, it was still believed that *TSPY* expression is strictly testis-specific. *TSPY* transcripts had been detected in testes of 22 and 26 week old human fetuses [7], and immunohistochemical analyses of adult human testes localized TSPY predominantly to spermatogonia and to a much weaker extent to primary spermatocytes [15]. The regulation of *TSPY* was still unknown, and only 1.2 kb immediately 5´ to the start of *TSPY* gene transcription had been identified as a potential promoter region that precedes all or almost all *TSPY* genes [5]. Tascou *et al*. [43] used 1.3 kb 5´-flanking region of a functional *TSPY* gene in an approach to express the SV40 TAg oncogene in early testicular germ cells. This fragment was initially analyzed *in vitro* in transiently transfected mouse spermatogonia derived GC-1 spg and mouse spermatocyte (between preleptotene and early pachytene stage) derived GC-4spc cell lines [44], and was shown to induce a higher transcriptional activity of a chloramphenicol transferase (CAT) reporter gene in GC-1spg cells in comparison to GC-4spc cells. This study was the first attempt to test a putative *TSPY* promoter fragment in transgenic mice *in vivo*. 

The authors were able to detect TAg transcripts via RT-PCR analyses not exclusively in the testis but also in the spleen, seminal vesicle, pituitary gland and adrenal gland of transgenic mice (Table 1). 

Thus, it seems that 1.3 kb 5´-region of a *TSPY* gene is insufficient in mediating a proper tissue-specific expression pattern in transgenic mice. However neither data about the transcription level nor about a proper translation of the transgene in the respective TAg expressing transgenic organs and tumors were reported [43]. Furthermore, no findings about the cellular testicular expression pattern of SV40 TAg driven by the used *TSPY* promoter fragment were published. 

#### 2.1.3. Expression of TAg failed to induce testicular germ cell tumors in transgenic mice but led to tumor formation in pituitary and adrenal glands and the seminal vesicles 

The transgenic mouse line *TSPY-TAg23* was originally generated to prove the potential of early male germ cells (spermatogonia) for malignant transformation, via targeted expression of SV40 large TAg, but large tumor antigen TAg failed to induce testicular germ cell tumors in transgenic mice [43]. Spermatogenesis was normal in *TSPY-TAg23* transgenic mice and neither *TSPY-TAg* transgenic founders nor male offspring of the line *TSPY-TAg23* developed testicular tumors [43]. This is probably due to the insusceptibility of mice for most testicular germ cell tumors, and the inherent strain differences in susceptibility. 

It is noteworthy that noninvasive pituitary adenomas that were originating from the anterior lobe occurred in approximately 65% of male and female offspring of the line *TSPY-TAg23*, and that also three other founder animals containing the *TSPY-TAg* transgene in their genome developed such tumors [43]. Approximately 20% of pituitary tumor cells expressed prolactin (PRL), while others expressed the adrenocorticotropic hormone (ACTH). Plasma PRL and corticosterone concentration were significantly increased in *TSPY-TAg* transgenic male and female mice in comparison to wild-type FVB mice. The strategy to induce targeted tumorigenesis by expression of SV40 large TAg under the control of 1.3 kb flanking sequences of the human *TSPY* gene led by the way to the generation of the first available mouse model for analyses of pituitary adenomas containing both, PRL and ACTH secreting tumor cells [43,45]. 31% of all mice with pituitary tumors (transgenic mice of the line *TSPY-TAg23* and one male* TSPY-TAg* transgenic founder) developed additionally tumors originating in the adrenal medulla. Tumorigenesis occurred also in a few older male progeny of founder 23 that developed a tumor of the seminal vesicle. All the different tumors derived independently due to the ectopic TAg expression in the respective organs. 

**Table 1:** Tissue specific expression pattern of mouse *TSPY-ps*, and the transgenes in the transgenic lines *TSPY-TAg23*, *Tg(TSPY-cre)33aYfcl* and *Tg(TSPY)9 Jshm.*

Expression analyses in:	1.3 kb *TSPY* promoter (*TSPY-TAg23)*	2.4 kb *TSPY* promoter *Tg(TSPY-cre)33aYfcl*	2.9 kb *TSPY* promoter *Tg(TSPY)9 Jshm*	*mouse TSPY-ps*
brain	-	+/#	+/++	+
pituitary gland	+	#*	n.a.	n.a.
testis	+	+/#	+/++/#	+
prostate	n.a.	-	+/--	-
seminal vesicle	+	-	n.a.	-
epididymis	n.a.	n.a.	+/--	n.a.
ovary	-	+/#	n.a.	n.a.
uterus	n.a.	-	n.a.	n.a.
lung	-	-	+/--	+
heart	-	-	+/--	+
spleen	+	+	+/--	+
liver	-	-	-/--	+
kidney	-	-	+	+
adrenal gland	+	n.a.	n.a.	n.a.
stomach	n.a.	n.a.	--	n.a.
large intestine	n.a.	n.a.	--	n.a.
thymus	n.a.	-	n.a.	+

n.a. not analyzed, detection of transgenic transcript via RT-PCR analyses (+) or Northern blot analyses (++), transcripts absent in RT-PCR analyses (-) or Northern blot analyses (--), transgene could be detected by immunostaining or expression of EGFP (#), no EGFP expression observed (#*). Data adapted from [16,43,47].

As several founders with different chromosomal integration sites of the transgene developed pituitary adenomas and adrenal medulla tumors it is unlikely that TAg expression in both glands is caused by a chromosomal position effect on transgene expression [46]. The expression of SV40 large and small T antigen (TAg) in both organs seems to be rather mediated by the used *TSPY* promoter fragment which directed an ectopic expression of TAg also in other organs than the testis. It was later shown that the 2.4 kb 5´region of a *TSPY* gene is not capable of inducing a Cre-recombinase reporter gene transcription in seminal vesicles of male *Tg(TSPY-cre)33aYfcl* and *Tg(TSPY-cre)33bYfcl* transgenic mice, and that 2.4 kb of the *TSPY* promoter is insufficient to induce a reporter gene expression (EGFP) in the anterior lobe of the pituitary gland in double transgenic *TSPY-Cre;Z/EG* mice (Table1; [16]). Especially the findings of the latter study clarified the inability of the 1.3 kb *TSPY* promoter fragment used to induce a proper tissue-specific expression pattern in transgenic mice. However, it cannot be ruled out that TSPY is also ectopically expressed in organs other than testis and prostate gland. The *TSPY* gene was recently classified as a tissue-enriched gene that is expressed at a much higher level in a specific organ (testis) than in other tissues [18], and further studies are needed to clarify the tissue-specific expression pattern of human *TSPY*.

### 2.2. The TSPY transgenic mouse line Tg(TSPY)9Jshm carrying a functional human TSPY gene

#### 2.2.1. The *TSPY* transgenic mouse line *Tg(TSPY)9Jshm*, an animal model closely resembles the organization and expression of human TSPY

**Figure 1:**
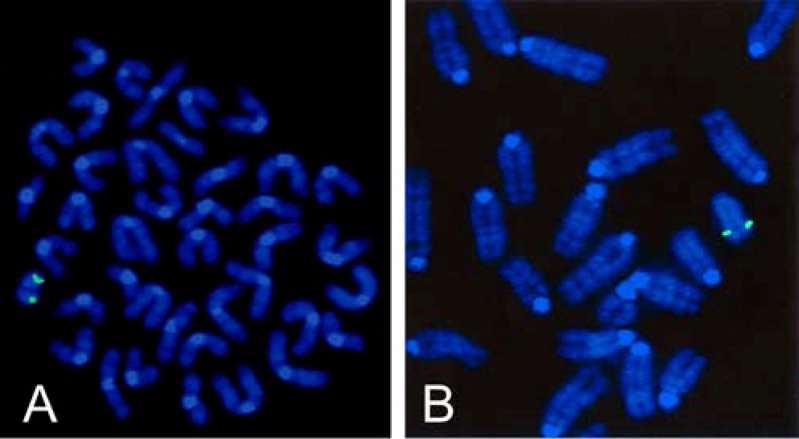
Fluorescence *In Situ* Hybridization analysis of a biotin-deoxyuridine 5-triphophate-labeled human *TSPY* probe (pHerbi) to DAPI counterstained chromosomes **(A, B)** of a *TSPY* transgenic adult mouse of the transgenic line *Tg(TSPY)9Jshm*. The recombinant plasmid pHerbi contains the human 8.2 kb genomic *TSPY* fragment that was used to generate the *TSPY* transgenic line *Tg(TSPY)9Jshm*[47]. Figure 1B was originally published in Schubert *et al.*, 2003 [47].

The putative oncogenic role of human TSPY in dysgenetic gonads and in testicular and prostatic tumor formation and also its function during fetal and adult male germ cell development could be much easier uncovered by the generation of a suitable animal model. With this in mind, repeated efforts finally culminated in only a single *TSPY* transgenic mouse line (*Tg(TSPY)9Jshm*) that was successfully established in 2003 by our group [47]. An 8.2 kb genomic fragment, containing a complete functional human *TSPY* gene (2796 bp), 2923 bp putative *TSPY* promoter region and 2481 bp 3´ region of human *TSPY* that was originally derived from the human cosmid cEMT [2] was microinjected in male pronuclei of fertilized oocytes of the outbred strain NMRI and led to the generation of 3 founding transgenic mice (two females and one male). We initially showed that this genomic fragment was correctly transcribed and spliced according to the human pattern in transiently transfected mouse spermatogonial derived GC-1 spg cells [48]. Although all founders were fertile, only the male founder (M9) transmitted the transgene to its progeny with paternal inheritance pattern. FISH and Southern blot analyses showed that approximately 50 copies of the human transgene are clustered at a single site at the distal part of the long arm of the mouse Y chromosome in the line *Tg(TSPY)9Jshm* (Figure 1). 

**Figure 2:**
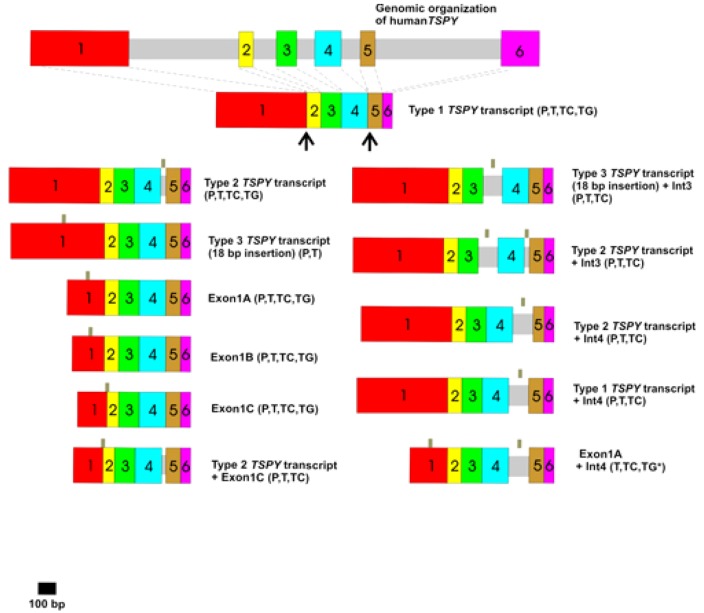
Schematic illustration of a human *TSPY* gene and the various *TSPY* transcripts in human prostatic (P, prostatic adenoma and benign prostatic hyperplasia) and testicular (T, human testis; TC, human testicular seminoma) tissues [17] and in mouse testes of the transgenic line *Tg(TSPY)9Jshm* (TG) [47,50,51]. Introns are shown as grey bars and exons indicated as colored boxes. Small grey bars above the splice variants indicate the use of alternative splice donor and/or acceptor sites. The *TSPY* cDNA region encoding the cyclin B binding domain (SET/NAP domain) is highlighted by a black line (aa residues 121-265) [22]. * [52]. Detailed descriptions of the differential spliced *TSPY* transcripts are given in reference [17]. Splice variants are termed in accordance to references [17] and [26]. 18 bp insertion: in frame insertion of GTG GAG CTG GTG GCG CAG within exon 1 of human *TSPY*, +: combination of different splicing patterns.

The similarity to the human *TSPY* organization in this transgenic line is a striking feature [2,3,5,49]. The transgene is primarily expressed in the testes, and mostly spliced in accordance with the human pattern [47]. In humans a *TSPY* transcription unit is 2.8 kb in length, is composed of 6 exons and 5 introns and generates a 1.3 kb main transcript, that codes for a polypeptide of 308 amino acids [5,17]. *TSPY* transcripts originating from the same *TSPY* transcription units are frequently alternatively spliced thereby producing various TSPY isoforms of yet unknown function. Up to now, 11 different *TSPY* splice variants that vary in sequence and length have been identified in prostatic, testicular and testicular tumor tissues [3,7,15,17,26] and many of these are also present in the testes of *TSPY* transgenic mice (Figure 2; [47,50,51]). 

All of these encode polymorphic TSPY isoforms harboring either the whole or part of the cyclin B binding SET/NAP domain of TSPY at their carboxyl termini [47,50,51,52]. Thus, this model can be useful in further studies for functional analyses of the different TSPY isoforms. It is yet uncertain whether the various different human *TSPY* transcripts originate from one or more specific or from all functional human *TSPY* transcriptional units, but this mouse model clearly demonstrates that many *TSPY* transcripts can be generated by one particular functional (though repetitive) *TSPY* transcription unit via alternative splicing. 

**Figure 3:**
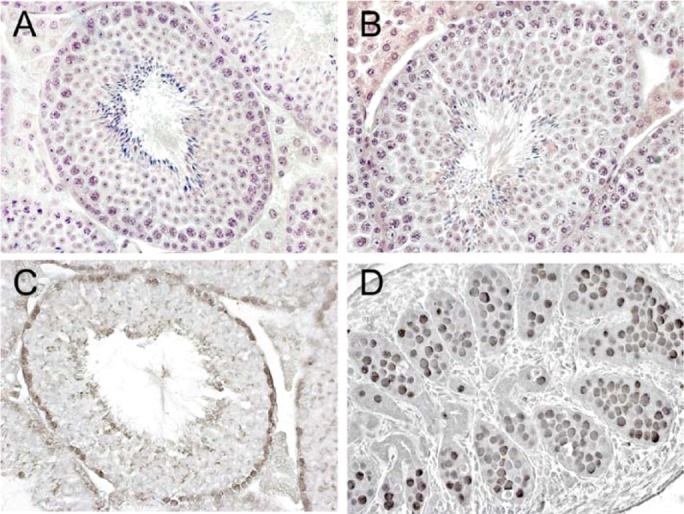
Testicular histology of fetal and adult *TSPY* transgenic mice (*Tg(TSPY)9Jshm*) and of an adult NMRI wildtype male. Hematoxylin and eosin staining of testis of an adult *TSPY* transgenic mouse **(A)** in comparison to an adult NMRI wildtype male **(B)**. Spermatogenesis and spermiogenesis of *TSPY* transgenic males were normal in comparison to wildtype mice. The TSPY specific antiserum 837/3 [15] immunostained spermatogonia and primary spermatocytes of the leptotene/zygotene stage in adult testes **(C)** and gonocytes and prespermatogonia in fetal gonads (**D**, testis of the embryonal stage 14.5 dpc) of mice of the line *Tg(TSPY)9Jshm*. A-D, orginal magnification x400. An unspecific immunostaining of the acrosome of round spermatids is also shown.

Northern blot analyses of different male organs (testis, epididymis, prostate, brain, lung, heart, stomach, large intestine, liver, spleen) showed that *TSPY* is expressed only in the testis and to a much lesser extent in the brain of adult transgenic males [47]. Using RT-PCR, the transgene has been shown to be transcribed at basal levels in other organs, such as the epididymis, prostate, kidney, lung, heart and spleen (Table1; [52]). Hence also 2923 bp 5´-flanking region of the *TSPY* gene are insufficient to induce a strictly testis and prostate specific expression pattern in transgenic mice. It was shown that the mouse* Tspy-ps* pseudogene, although being non-functional, is transcribed most prominently in the testis, but also at lower levels in other organs, such as the brain, thymus, lung, heart, liver, spleen and kidney (Table 1; [16]). Thus, the TSPY transgenic expression pattern mimics partially that of the murine counterpart. It is also conceivable that trans-acting factors mediating a proper *TSPY* expression are less conserved in human and mice. Although the tissue specific expression pattern of the human transgene does not exactly reflect the human pattern, the *TSPY* transgene is properly regulated within the fetal, neonatal and adult testis [47,52]. As in humans, the *TSPY* transgene is mainly expressed in spermatogonia and in primary spermatocytes at the preleptotene, leptotene, and zygotene stage in postnatal testes [47,50,51,52,53], and in gonocytes and prespermatogonia in fetal testes (Figure 3; [14]). 

The testicular expression pattern and the cellular topology of the TSPY** transgene within testes of the different ontogenetic stages seem to be nearly in total accordance with the human situation. These observations suggest that the line *Tg(TSPY)9Jshm* could be useful for further analyses of the regulation and expression of human TSPY. 

Initial studies demonstrate that *TSPY* transcription levels can be raised by androgene administration in androgen-responsive human prostate cancer cell line LNCaP, suggesting that TSPY expression could be regulated by androgens and its receptor within the testis and the prostate [54,55]. Nonetheless, another study has shown that *TSPY* transcription is upregulated in androgen-independent LNCaP-C81 prostate cancer cells in comparison to androgen-dependent LNCaP-C33 cells [56]. As androgens play an important role in the development and differentiation of the human prostate and in prostatic tumorigenesis, and TSPY expression is elevated in prostate cancers of increasing Gleason grades, one may speculate that TSPY exerts an oncogenic or tumor predisposition role in prostate cancer formation [17,55]. If this is mediated by an aberrant and/or androgen-responsive TSPY expression in epithelial cell of the native prostate and/or tumor cells of prostate cancers remain to be elucidated. As the human *TSPY* transgene is also expressed and correctly spliced in the prostate of transgenic mice of the line *Tg(TSPY)9Jshm* [52], this mouse model could be also useful to address these issues.

#### 2.2.2. A suitable transgenic mouse model for functional analyses of TSPY in male fetal and adult germ cells 

**Figure 4:**
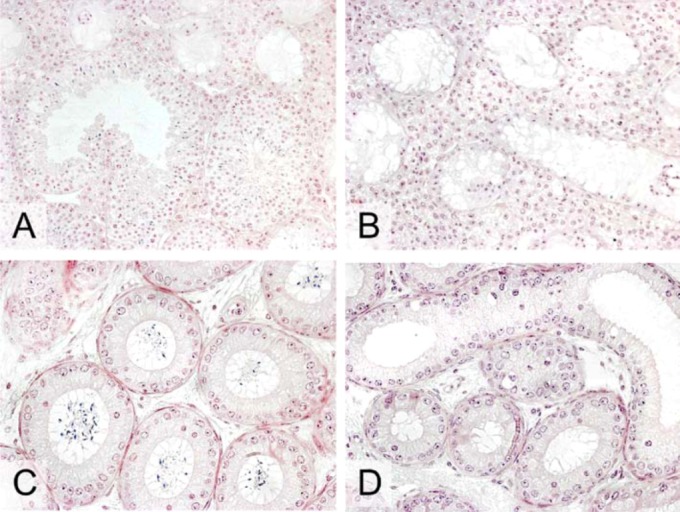
Histological haematoxylin and eosin stained sections of testes (**A**,**B**, orginal magnification x200) and epididymides (**C**,**D**, orginal magnification x400) of adult B6;NMRI*-Kit* *^W-v^/Kit* *^W-v ^Tg(TSPY)* (A,C) and B6;NMRI*-Kit* *^W-v^/Kit* *^W-v^* male mice (B,D). While spermatogenesis arrests at the spermatogonial stage in the non transgenic B6;NMRI*-Kit* *^W-v^/Kit* *^W-v^* testis (B) and no sperm are present in the respective epididymis, a partial rescue of spermatogenesis and spermiogenesis is shown in the *TSPY* transgenic Kit-deficient testis (A). The respective epididymis is also filled with sperm (C).

*TSPY* transgenic NMRI male mice of the line *Tg(TSPY)9Jshm* are phenotypically normal and fertile and spermatogenesis is neither impaired nor enhanced by the *TSPY* transgene (Figure 3) [47]. This is not surprising because it is likely that one or more as yet unknown gene(s) have taken over a TSPY analogous function within the mouse testis. One or more of the five known *Tspy-like* autosomal genes in the mouse genome could be candidates [49], and knock-out experiments performed on different autosomal *Tspy-like* genes would be a sensible attempt to elucidate their biological functions also within the testis. It is conceivable that spermatogenesis is not affected by the *TSPY* transgene due to the fact that the transgenic protein and its functional analog both fulfill the same functions within male germ cells, and thereby recognize the same partners. Hence, TSPY transgenic effects on germ cell development might become apparent when spermatogenesis proceeds under suboptimal conditions [47]. Schöner *et al.* [50] addressed the issue of whether TSPY is capable of restoring the spermatogenic failure of KIT-deficient *Kit^W-v^/Kit^W-v^* mutants and demonstrated a significant partially rescue of spermatogenesis, spermiogenesis and fertility in *TSPY* transgenic B6;NMRI*-* *Kit^W-v^/Kit^W-v^* male mice in comparison to controls (Figure 4). 

Although these data point to a role of TSPY in fetal and adult germ cell proliferation, it is still unknown how TSPY supports the germ cell depleted spermatogenesis in the KIT deficient testis. The observed restoration of spermatogenesis could be due to a proliferative or apoptosis protective effect of the transgene in fetal or postnatal germ cell development. These findings suggest the line *Tg(TSPY)9Jshm* as a suitable animal model for further studies to clarify TSPY putative functions in fetal and adult male germ cell development.

#### 2.2.3. The line *Tg(TSPY)9Jshm* is insufficient to elucidate TSPY role in gonadoblastoma formation and testicular tumorigenesis

It should be mentioned here that *TSPY* transgenic mice of the line *Tg(TSPY)9Jshm* are not susceptible to any somatic or gonadal tumors [47,50,51]. The insusceptibility of the laboratory mouse for most testicular type-II germ cell tumors and their precursor stages questions the suitability of a *TSPY* transgenic mouse model as a tool to address TSPY putative oncogenic function in gonadal tumor formation. Testicular germ cell tumors in mice are nearly restricted to teratomas and embryonal carcinomas [40,41,42,57]. To the best of our knowledge only one transgenic mouse line that overexpressed the glia cell line derived neurotrophic factor (GDNF) promotes the formation of seminomatous tumors that mimics human seminomas [58]. Besides, both H-RasV12 transfected and cyclin D2 and cyclin E1 cotransfected mouse spermatogonial stem cells (SSCs) are capable of inducing placental alkaline phosphatase (PLAP) expressing germ cell tumors in recipient mouse testes [59]. On the other hand, it was demonstrated that a female founder harboring a 12.5 kb *TSPY* transgene, including also a complete 2.8 kb structural *TSPY* gene, developed gonadoblastoma-like structures in both ovaries, which expressed the *TSPY* transgene in a pattern resembling the expression pattern of TSPY in human gonadoblastoma [49,53]. In humans, gonadoblastoma arises in undifferentiated gondal tissue within dysgenetic gonads of intersex patients or phenotypic females carrying at least a part of Y chromosome that includes the GBY locus with an estimated risk of up to 30% [29,60], and human *TSPY* is at the moment the most likely candidate gene for GBY. The interesting finding that TSPY is able to induce gonadoblastoma-like structures in gonads of a female *TSPY* transgenic mouse [49,53] would indeed strengthen *TSPY* candidacy for GBY. As the *TSPY* transgenic line *Tg(TSPY)9Jshm* would never transmit the transgene to a female mice, the generation of a second *TSPY* transgenic line, with an autosomal or X-chromosomal integration site, that allows a proper expression of the *TSPY* transgene in the female gonad was one of our goals. 

### 2.3. Transgenic lines Tg(TSPY-cre)33aYfcl and Tg(TSPY-cre)33bYfcl: A Cre-recombinase transgene under direction of a 2.4 kb TSPY promoter fragment

#### 2.3.1. kb human *TSPY* promoter fragment is insufficient to mediate a proper tissue-specific expression pattern of a reporter gene in transgenic mice

The first attempt to prove that a *TSPY* promoter fragment can be active in a female gonad was published in 2005 by Kido and Lau [16]. Two founder mice (one male and one female, both of B6CBAF1 origin) were generated by microinjection of a Cre-recombinase transgene driven by a 2383 bp putative *TSPY* promoter region and 43 bp from the transcription start site of a human *TSPY* construct that was derived from the plasmid, pTSPY12.5 [7]. Only the female founder transcribed and transmitted the transgene properly, and generated—due to two different autosomal integration sites—two different Cre recombinase transgenic lines with different transgenic copy numbers, designated as *Tg(TSPY-cre)33aYfcl* (42 copies) and *Tg(TSPY-cre)33bYfcl* (six copies). Both lines showed a similar tissue specific expression pattern but differed in expression strength of the Cre-recombinase transgene. Due to higher expression levels of the transgene in the line *Tg(TSPY-cre)33aYfcl*, this line was characterized in much more detail and will be discussed in this review. 2383 bp putative *TSPY* promoter region induced transgenic transcripts prominently in the gonads (testes and ovaries) and the brain (cerebral cortex and cerebellum) and to a lesser extent in the spleen but not in other organs, such as seminal vesicle, prostate, lung, heart, liver, kidney, thymus and uterus (Table1; [16]). Especially the expression of the Cre-recombinase transgene in the brain (cerebellum and cerebral cortex) of male and female mice of the line *Tg(TSPY-cre)33aYfcl* [16] and the detection of human *TSPY* transgenic transcripts in the brain (cerebrum) of mice of the line *Tg(TSPY)9Jshm* [47] are both striking findings, and the availability of TSPY ESTs derived from the medulla of human brains (BX281192, B1828033) in databases [16] could point to an up to now unexpected role of TSPY in the male brain. Because the *TSPY*-Cre-recombinase transgene expression pattern mirrors widely that of the human ortholog, 2.4 kb of the putative *TSPY* promoter seem to be sufficient to direct a proper tissue-specific expression pattern of a *TSPY*-Cre recombinase transgene.

#### 2.3.2. kb promoter of human *TSPY* is sufficient to direct the expression of a reporter gene in oocytes but failed to induce a proper testicular expression pattern

**Figure 5:**
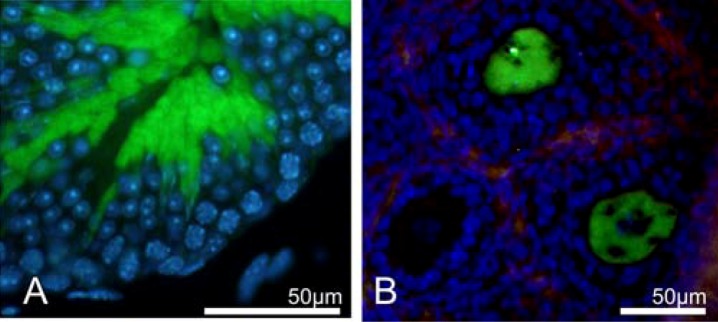
Expression of EGFP in testis **(A)** and ovary **(B)** of double transgenic *TSPY-Cre;Z/EG* mice [16]. DAPI (blue) counterstained the DNA and EGFP fluorescence is shown in green. Within the testis (A), EGFP is exclusively expressed in round and elongated spermatids (stained in green), but not in other germ cells or somatic cells within the testis (stained blue). In B, an immunofluorescence image of an ovary from an 8-day old *TSPY-Cre;Z/EG* mice is shown. EGFP is expressed exclusively in maturing oocytes (stained green), while granulosa cells within the ovary were EGFP negative (stained blue). Figure 5 is published with the acknowledgement of Tatsuo Kido as the source.

Double transgenic *TSPY-Cre;Z/EG* mice were generated by crossing mice of the line *Tg(TSPY-cre)33aYfcl* with a Z/EG reporter line [61]. These *TSPY-Cre;Z/EG* mice expressed EGFP specifically after Cre recombinase-mediated action. The 2.4 kb fragment of the human *TSPY* promoter was capable of producing green fluorescence in testicular (round and elongated spermatids) and ovarian (oocytes of late primary and secondary follicles) germ cells (Figure 5) and in neurons of the central and peripheral nervous system in adult double transgenic mice [16]. 

A remarkable finding of this study is that a 2.4 kb promoter of human *TSPY* is sufficient to direct the expression of a reporter gene in ovarian oocytes. Cre-recombinase transcripts were detected in ovaries of the embryonal stage E12.5, of newborn and in ovaries of prepubertal transgenic *Tg(TSPY-cre)33aYfcl* females but EGFP expression in double transgenic mice was not observed in primary oocytes, suggesting that the used promoter fragment is more active at late meiotic stages [16]. The activity of a 2.4 kb human *TSPY* promoter in an ovarian environment is of great importance, and supports the hypothesis that *TSPY* is indeed GBY [16,53]. 

The aberrant testicular expression pattern of adult double transgenic mice (Figure 5) in comparison to that observed in humans and in transgenic males of the line *Tg(TSPY)9Jshm* is astonishing, and could partially be due to spermatogonial and spermatocytic enhancer elements that are present within the first 540 nucleotides at the 5´end of the used 2923 bp putative *TSPY* promoter region in line *Tg(TSPY)9Jshm* [47]. Further *TSPY* promoter analyses are indispensable to confirm this assumption. It was also shown by RT-PCR analyses that *Cre* transgenic transcripts are present in embryonal stage E12.5, newborn and subadult transgenic mice testes at time points when germ cells have not yet undergone second meiotic division [16]. These observations point to an insufficient expression level of the transgene in early spermatogenic germ cells. It is noteworthy that the 2.4 kb *TSPY* promoter fragment also directed expression of the reporter gene in elongated spermatids similar to the testicular expression pattern of rat *Tspy* [62], and it is conceivable that the mouse preserved still parts of the rodent *Tspy* expression pattern by expressing the Cre-recombinase transgene preferentially in spermatids. On the other hand, the findings that *TSPY* is not expressed in elongated spermatids in humans [15,16] and in transgenic mice of the line *Tg(TSPY)9Jshm* [47] do not support the notion that laboratory mouse perpetuates the testicular expression pattern of rat Tspy. As both lines, *Tg(TSPY-cre)33aYfcl* and* Tg(TSPY-cre)33bYfcl*, with the transgene integrated at different chromosomal site, do not differ with respect to their EGFP testicular expression pattern, the influence of a chromosomal position effect on testicular expression can be excluded [46]. Finally, it can also not be ruled out that the observed differences in testicular expression in the transgenic lines *Tg(TSPY-cre)33aYfcl*,* Tg(TSPY-cre)33bYfcl* and *Tg(TSPY)9Jshm* could also be due to genetic background differences of the used mouse strains. Fertility of the EGFP expressing male and female double transgenic mice was normal and not affected by the presence of either transgene.

An intriguing finding of the adult *TSPY*-Cre;Z/EG mice was the neuron specific EGFP expression in brain, including the cerebellum, cerebral cortex and hippocampus. It is also noteworthy that EGFP was expressed in the optic cord and the trigeminal nerve of the brain stem but could not be detected in the anterior lobe of the pituitary gland in adult mice [16]. At embryonal stages E12.5-13.5, EGFP is specifically expressed in the trigeminal ganglia, the trigeminal nerve and in neurons of the dorsal root ganglia. These findings raise the question whether the *TSPY* promoter is also active in human neurons, and whether TSPY is able to fulfill yet unknown function in neural development and within the brain. 

## 3. Conclusions and outlook

The functions of human *TSPY* genes are still unknown, but multiple *in vivo* and *in vitro* studies point to a role of TSPY in cell cycle regulation of fetal and spermatogenic germ cells. Especially the multiplicity and sequence diversity of human *TSPY* gene copies make the analyses of regulation, expression and function extremely difficult. The identification of a non- functional murine single-copy *Tspy-ps* pseudogene only in species of the subgenus *Mus*, including the laboratory mouse, enabled the generation of different transgenic mouse models as *in vivo* models for functional, regulative and expression analyses of human TSPY. Despite multiple efforts by several groups, only three classical transgenic mouse models have been successfully established, although much more founding transgenic mice have been generated. Overall, 10 founder mice were generated, harboring a complete 2.8 kb structural *TSPY* gene and 2.4 or 2.9 kb *TSPY* promoter region, but only one male founder transmitted the transgene with paternal inheritance pattern [47,49]. It indeed seems that *TSPY* transgenic mice are difficult to establish [49], and it is nevertheless tempting to speculate that a Y chromosomal transgenic integration is essential for a successful transgenic mouse model. It is also conceivable that *TSPY* transgenes with autosomal or X-chromosomal integration site could be dysregulated, and that an ectopic expression of TSPY in other organs than the testis and prostate is harmful for the host [49]. The mouse *Tspy-ps* pseudogene could be used in future experiments as a targeting site to “smuggle” a human *TSPY* gene under the dependence of a tissue specific promoter on the mouse Y chromosome. A comparable targeting strategy for a Y-specific integration of a transgene was already successfully established in 2005 [63]. A murine myostatin (*MSTN*) coding sequence under the control of a rat myosin light chain 1 F (*MLC1F*) promoter and 1/3 enhancer for expression in skeletal muscle was introduced on the mouse Y chromosome in gene-targeted mice [63]. 

With the help of three transgenic mouse lines harboring reporter genes or a complete human *TSPY* gene driven by 1.3 kb [43], 2.4 kb [16] and 2.9 kb [47] of the *TSPY* promoter, it was shown that 2.9 kb of the human *TSPY* promoter is sufficient to direct a proper testis specific expression pattern of a transgene in transgenic mice. Especially the latter model can serve as an animal model to contribute significantly to our understanding of TSPY regulation, expression and function, and could be useful to address TSPY putative oncogenic role in prostatic and testicular tumor formation in future studies. Besides the transgenic mouse line *TSPY-TAg23* represents a useful tumor mouse model for pituitary adenomas secreting PRL and ACTH [43]. The different testicular expression pattern of the transgenic lines *Tg(TSPY-cre)33aYfcl*,* Tg(TSPY-cre)33bYfcl* and Tg*(TSPY)9Jshm* (Table 1) could in general be influenced by the different length of the promoter, the integration sites of the transgenes within the mouse genome, and the genetic background of the hosts [49]. As only one *TSPY* transgenic mouse line has been successfully established, the generation of further *TSPY* transgenic lines with Y-chromosomal or autosomal integration sites must be a desirable main aim. Especially the interesting finding that TSPY is able to induce gonadoblastoma-like structures in gonads of a female *TSPY* transgenic mouse supports the hypothesis that *TSPY* could be indeed GBY. Generation of a second *TSPY* transgenic line, with an autosomal or X-chromosomal integration site, that allows a proper expression of the *TSPY* transgene in ovaries should be a main aim of future studies. 

As the genetic background of a mouse model can dramatically impact on its phenotype [34,50,51], the choice of the proper mouse strain for transgenesis is important to avoid the pitfall of time-consuming backcrossing into the genetic background of choice. 

All these findings raise the question, whether alternative animal models could be utilized in future studies aimed at investigating regulation, expression and function of TSPY. As the testicular expression pattern of rat Tspy does not properly mimic that of human *TSPY*, a targeted gene knock-out of rat *Tspy* would only partially be useful to uncover the TSPY function in humans. It is supposed that rat Tspy** could act as histone chaperone in elongated spermatids, by the time core histones are replaced by basic protamines [62]. The fact that rat and human TSPY can both interact *in vitro* with core histones suggest that both direct similar functions in spermiogenesis [62]. Especially the recent developed zinc finger nuclease technology which was also successfully applied to the rat [64,65] could be suitable for the generation of targeted *rTspy* gene disruption. The zinc finger nuclease technology seems to be in general a promising tool to generate knock-out mutations at specific loci in mammalians and will offer new opportunities for *Tspy* gene knock-outs in other rodent species. Finally knock-out experiments performed on each of the six different *Tspy-like* genes would be a feasible approach to elucidate their biological functions also within the testis.
